# Mining the Characteristics of COVID-19 Patients in China: Analysis of Social Media Posts

**DOI:** 10.2196/19087

**Published:** 2020-05-17

**Authors:** Chunmei Huang, Xinjie Xu, Yuyang Cai, Qinmin Ge, Guangwang Zeng, Xiaopan Li, Weide Zhang, Chen Ji, Ling Yang

**Affiliations:** 1 Department of Geriatrics Xinhua Hospital Shanghai Jiaotong University School of Medicine Shanghai China; 2 Department of Emergency Xinhua Hospital Shanghai Jiaotong University School of Medicine Shanghai China; 3 School of Public Health Shanghai Jiao Tong University School of Medicine Shanghai China; 4 The Health Center of Nansheng Town Wuzhishan China; 5 Center for Disease Control and Prevention, Pudong New Area Shanghai China; 6 Pudong Institute of Preventive Medicine, Pudong New Area Fudan University Shanghai China; 7 Big Data and Artificial Intelligence Center Zhongshan Hospital Fudan University Shanghai China; 8 Warwick Clinical Trials Unit Warwick Medical School Coventry United Kingdom

**Keywords:** SARS-CoV-2, COVID-19, coronavirus disease, social media, Sina Weibo, help

## Abstract

**Background:**

In December 2019, pneumonia cases of unknown origin were reported in Wuhan City, Hubei Province, China. Identified as the coronavirus disease (COVID-19), the number of cases grew rapidly by human-to-human transmission in Wuhan. Social media, especially Sina Weibo (a major Chinese microblogging social media site), has become an important platform for the public to obtain information and seek help.

**Objective:**

This study aims to analyze the characteristics of suspected or laboratory-confirmed COVID-19 patients who asked for help on Sina Weibo.

**Methods:**

We conducted data mining on Sina Weibo and extracted the data of 485 patients who presented with clinical symptoms and imaging descriptions of suspected or laboratory-confirmed cases of COVID-19. In total, 9878 posts seeking help on Sina Weibo from February 3 to 20, 2020 were analyzed. We used a descriptive research methodology to describe the distribution and other epidemiological characteristics of patients with suspected or laboratory-confirmed SARS-CoV-2 (severe acute respiratory syndrome coronavirus 2) infection. The distance between patients’ home and the nearest designated hospital was calculated using the geographic information system ArcGIS.

**Results:**

All patients included in this study who sought help on Sina Weibo lived in Wuhan, with a median age of 63.0 years (IQR 55.0-71.0). Fever (408/485, 84.12%) was the most common symptom. Ground-glass opacity (237/314, 75.48%) was the most common pattern on chest computed tomography; 39.67% (167/421) of families had suspected and/or laboratory-confirmed family members; 36.58% (154/421) of families had 1 or 2 suspected and/or laboratory-confirmed members; and 70.52% (232/329) of patients needed to rely on their relatives for help. The median time from illness onset to real-time reverse transcription-polymerase chain reaction (RT-PCR) testing was 8 days (IQR 5.0-10.0), and the median time from illness onset to online help was 10 days (IQR 6.0-12.0). Of 481 patients, 32.22% (n=155) lived more than 3 kilometers away from the nearest designated hospital.

**Conclusions:**

Our findings show that patients seeking help on Sina Weibo lived in Wuhan and most were elderly. Most patients had fever symptoms, and ground-glass opacities were noted in chest computed tomography. The onset of the disease was characterized by family clustering and most families lived far from the designated hospital. Therefore, we recommend the following: (1) the most stringent centralized medical observation measures should be taken to avoid transmission in family clusters; and (2) social media can help these patients get early attention during Wuhan’s lockdown. These findings can help the government and the health department identify high-risk patients and accelerate emergency responses following public demands for help.

## Introduction

### Background

In December 2019, pneumonia cases of unknown origin were reported in Wuhan City, Hubei Province, China. The illness was identified and officially named as coronavirus disease 2019 (COVID-19), which is caused by a novel viral strain called severe acute respiratory syndrome coronavirus 2 (SARS-CoV-2) [1-3] and resembles severe acute respiratory syndrome coronavirus (SARS-CoV) [4]. Since the outbreak, COVID-19 has spread rapidly. Person-to-person transmission in hospital and family settings had occurred due to close contact [5,6]. On January 23, 2020, Wuhan shut down public transportation and was placed under lockdown, and residents were not allowed to leave the city. As of February 20, 2020, the accumulative number of laboratory-confirmed patients in Wuhan was 45,346. The health care system was further overburdened as patients with mild symptoms sought hospitalization instead of self-isolation, mainly due to the anxiety and panic instigated by the epidemic [7]. After failing to be admitted to a hospital, patients sought help on Sina Weibo, a Chinese microblogging site similar to Twitter that allows people to communicate and share information instantly [8]. Social media has become an important channel for promoting risk communication during the crisis [9,10] and can be used to measure public attention given to public health emergencies [11], such as H7N9 [12-14], Ebola [9,15-19], Zika virus [10,20,21], Middle East respiratory syndrome (MERS-CoV) [22], and Dengue fever [23].

Since the COVID-19 outbreak, social media, especially Sina Weibo, has become an important platform for the public to obtain epidemic-related information quickly and effectively. According to the official outbreak data released by Sina Weibo on February 26, 2020, 51.2 million users cumulatively posted 350 million pieces of epidemic-related content. Online readership of epidemic-related topics reached 754.5 billion. Sina Weibo established a communication channel that allowed the government to effectively listen and respond to public opinion quickly. Here, by collecting data from Sina Weibo from February 3 to 20, 2020, we aim to analyze the characteristics of suspected or laboratory-confirmed patients with the SARS-CoV-2 infection.

### Objective

In this study, we describe the characteristics of suspected or laboratory-confirmed patients with the SARS-CoV-2 infection, the distribution of patients throughout Wuhan, and the relationship between helpers (eg, relative, friend, spouse, sibling) and patients. Social media was used to obtain timely access to public demand so that the government and the health department could identify high-risk patients and take measures to help these patients.

## Methods

### Overview

**Figure 1:**
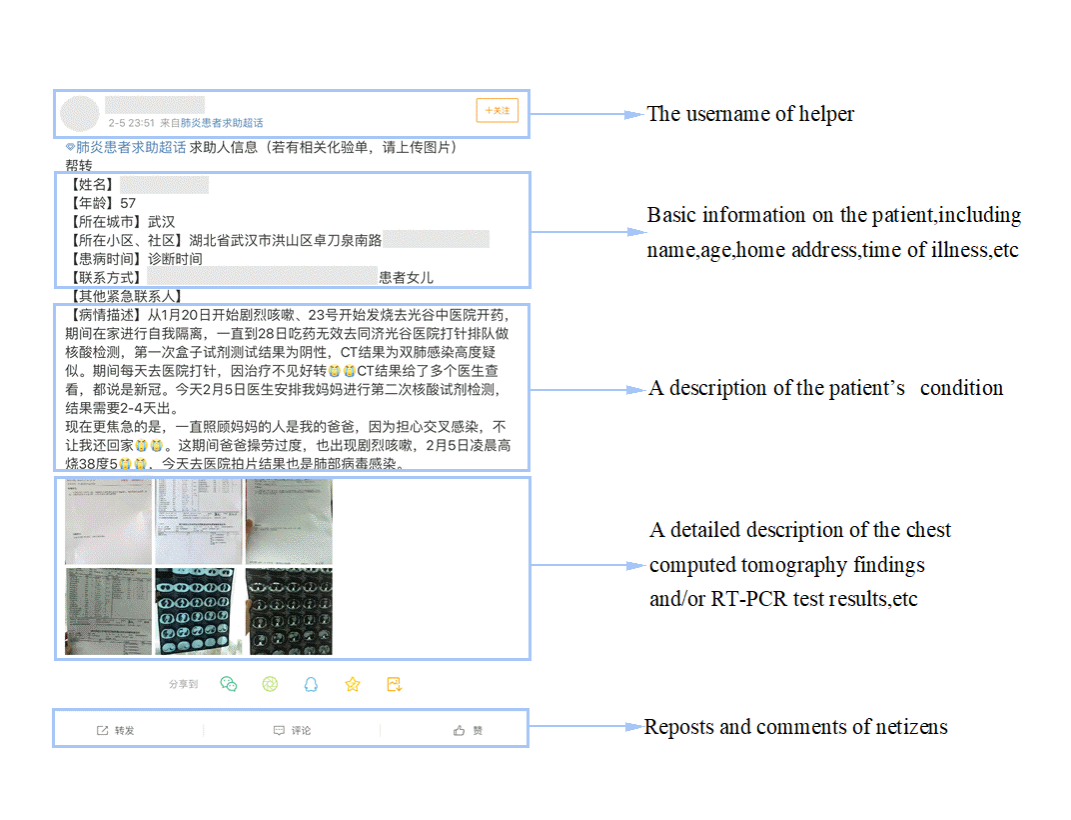
An example of a patient with coronavirus disease (COVID-19) seeking help on Sina Weibo. RT-PCR: reverse transcription-polymerase chain reaction.

**Figure 2:**
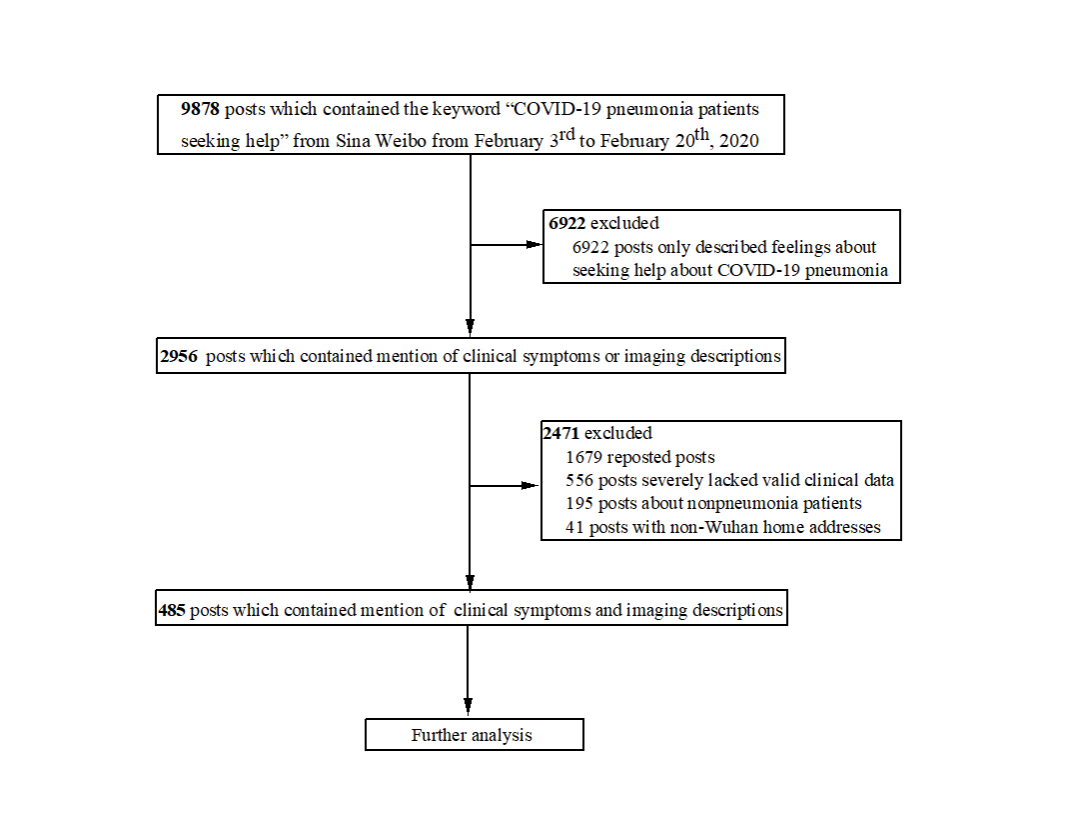
Study flow diagram. COVID-19: coronavirus disease.

Sina Weibo launched a platform to provide online help channels for patients infected with SARS-CoV-2. From February 3 to 20, 2020, we obtained 9878 posts by using the keyword 肺炎患者求助 (COVID-19 pneumonia patients seeking help) from Sina Weibo through its application programming interface (API). Python (Python Software Foundation) was used to implement a rule-based screening and classification method on the PyCharm platform. We used the collected posts as a training set, including related posts and unrelated posts. Based on the post-for-help rules formulated by Sina Weibo, we considered the post text, as well as keywords pertaining to name, age, home address, time of illness, and description of illness as a related post; otherwise, it was deemed an irrelevant post. We excluded 6922 irrelevant posts that only described opinions and feelings about help seeking related to COVID-19 and initially collected 2956 related posts that contained mentions of clinical symptoms and/or imaging descriptions. Then, we manually screened out and excluded posts. We excluded 1679 reposted posts, 556 posts with a significant amount of missing valid clinical data, 195 nonpneumonia patient posts, and 41 patient posts with non-Wuhan home addresses. Finally, we selected 485 patient posts that presented clinical symptoms and imaging descriptions (Figures 1 and 2). The number of patient posts on Sina Weibo has been declining because these patients have actively deleted posts upon hospital admission.

We collected clinical symptoms, chest computed tomography (CT) findings (the chest CT was only summarized for those who provided a clinical report), days from illness onset to online help, days from illness onset to RT-PCR testing, RT-PCR test results, the relationship between helpers and patients, and home address details from Sina Weibo’s records. We performed a study on the clinical characteristics of suspected or laboratory-confirmed patients with the SARS-CoV-2 infection seeking help on Sina Weibo. Suspected cases were identified as having fever or respiratory symptoms such as shortness of breath, cough, productive sputum, or chest pain. A laboratory-confirmed case with SARS-CoV-2 infection was defined as a positive result to high throughput sequencing or real-time reverse transcription-polymerase chain reaction (RT-PCR) assay of throat swabs and sputum [2].

We also used a descriptive research methodology to analyze the distribution of patients throughout Wuhan and the relationship between helpers and patients. The distance from patients' home to the nearest designated hospital was calculated using the geographic information system ArcGIS. The data used in the current study is publicly accessible on Sina Weibo and readers can obtain the raw data online [24]. We have effectively protected the privacy of subjects and strictly adhered to the principle of confidentiality in terms of information collection, storage and transmission, and information use and deletion. The study was approved by the Shanghai Jiaotong University Xinhua Hospital Ethics Committee and was carried out in accordance with the Declaration of Helsinki. We have made an application for exemption from informed consent and obtained approval.

### Statistical Analysis

Continuous variables were expressed as median (IQR) when appropriate. Categorical variables were summarized as counts and percentages in each category. Analysis was conducted using SPSS, version 19.0 (IBM). We used ArcGIS, version 10.2.2, to plot the numbers of patients seeking help on a map.

## Results

### Demographic and Clinical Characteristics

We selected 485 patients with suspected or laboratory-confirmed SARS-CoV-2 infection with at least clinical symptoms and imaging descriptions from Sina Weibo. The demographic and clinical characteristics were shown in Table 1. The median age was 63.0 years (IQR 55.0 to 71.0), 0.21% (1/470) of patients were below 15 years of age, and 50.10% (243/485) were female. Fever (408/485, 84.12%) was the most common symptom. Other symptoms reported by patients included fatigue (224/485, 46.19%), shortness of breath (261/485, 53.81%), nausea or vomiting (81/485, 16.70%) and diarrhea (61/485, 12.58%). In total, 23.09% (112/485) of patients had at least one underlying disorder (eg, hypertension, chronic obstructive pulmonary disease, etc). All patients underwent chest CT. Of these patients, 35.26% (171/485) reported lung infection on the chest CT but did not provide their clinical reports. In the remaining 64.74% (314/485) of patients, the most common pattern on chest CT was ground-glass opacity (237/314, 75.48%) and bilateral patchy shadowing (191/314, 60.83%). The median time from illness onset to RT-PCR testing was 8.0 days (IQR 5.0-10.0), and the median time from illness onset to online help was 10.0 days (IQR 6.0-12.0). RT-PCR testing was performed in 52.16% (253/485) of patients; 68.38% (173/253) were positive, 1.98% (5/253) were suspected cases, and 10.67% (27/253) were negative.

**Figure 3:**
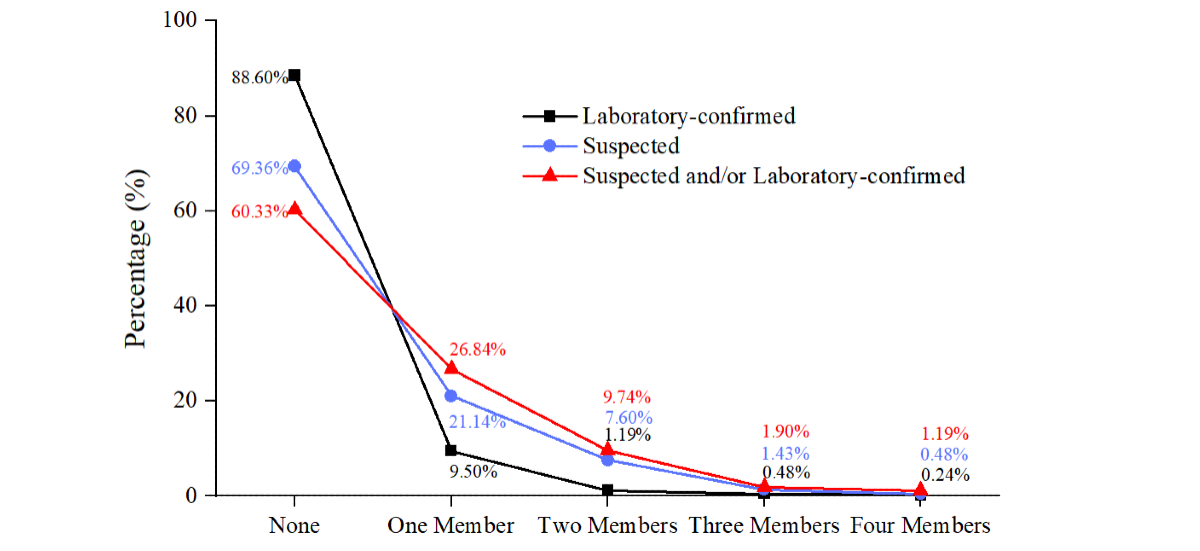
The distribution of family clusters.

The 485 patients came from 421 families, and 39.67% (167/421) of these families had at least one family member with a laboratory-confirmed and/or suspected diagnosis of SARS-CoV-2; 11.40% (48/421) of families had one laboratory-confirmed family member only. Families with one confirmed case accounted for 9.50% (40/421); two, three, and four confirmed members accounted for 1.19% (5/421), 0.48% (2/421), and 0.24% (1/421), respectively. A suspected diagnosis occurred in 30.64% (129/421) of families; a family with one suspected member accounted for 21.14% (89/421), two suspected members accounted for 7.60% (32/421), three suspected members accounted for 1.43% (6/421), and four suspected members accounted for 0.48% (2/421) (Figure 3).

**Table 1:** Clinical characteristics of suspected or laboratory-confirmed patients with severe acute respiratory syndrome coronavirus 2 (SARS-CoV-2) infection (N=485).

Characteristic		Value
Age (years), median (IQR)		63.0 (55.0-71.0)
**Age groups (n=470), n (%)**		
	0-14 years	1 (0.21)
	15-49 years	74 (15.74)
	50-64 years	178 (37.87)
	≥65 years	217 (46.17)
Sex (female; n=485), n (%)		243 (50.10)
**Respiratory symptoms, n (%)**		
	Fever (temperature ≥37.3℃)	408 (84.12)
	Cough	190 (39.18)
	Fatigue	224 (46.19)
	Shortness of breath	261 (53.81)
	Nausea or vomiting	81 (16.70)
	Diarrhea	61 (12.58)
**Coexisting disorders (n=485), n (%)**		
	Any	112 (23.09)
	Chronic obstructive pulmonary disease	10 (2.06)
	Diabetes	43 (8.87)
	Hypertension	55 (11.34)
	Coronary heart disease	38 (7.84)
	Cerebrovascular diseases	12 (2.47)
	Cancer^a^	7 (1.44)
	Chronic renal diseases	7 (1.44)
	Immunodeficiency	2 (0.41)
	Hepatitis B infection^b^	3 (0.62)
**Radiologic findings: abnormalities on chest CT** ^**c**^ **(n=314), n (%)**		
	Ground-glass opacity	237 (75.48)
	Local patchy shadowing	20 (6.37)
	Bilateral patchy shadowing	191 (60.83)
	Interstitial abnormalities	6 (1.91)
Days from illness onset to online help, median (range)		10 (6-12)
Days from illness onset to RT-PCR^d^ testing, median (range)		8 (5-10)
**Underwent RT-PCR testing (n=253), n (%)**		253 (52.16)
	Positive	173 (68.38)
	No result	48 (18.97)
	Suspect	5 (1.98)
	Negative	2 (10.67)

^a^Cancer referred to any malignancy.

^b^Hepatitis B infection denotes a positive test for hepatitis B surface antigen, with or without elevated alanine or aspartate aminotransferase levels.

^c^CT: computed tomography.

^d^RT-PCR: reverse transcription-polymerase chain reaction.

### The Distribution of Patients Throughout Wuhan and the Distance Between Helpers and Hospitals

**Figure 4:**
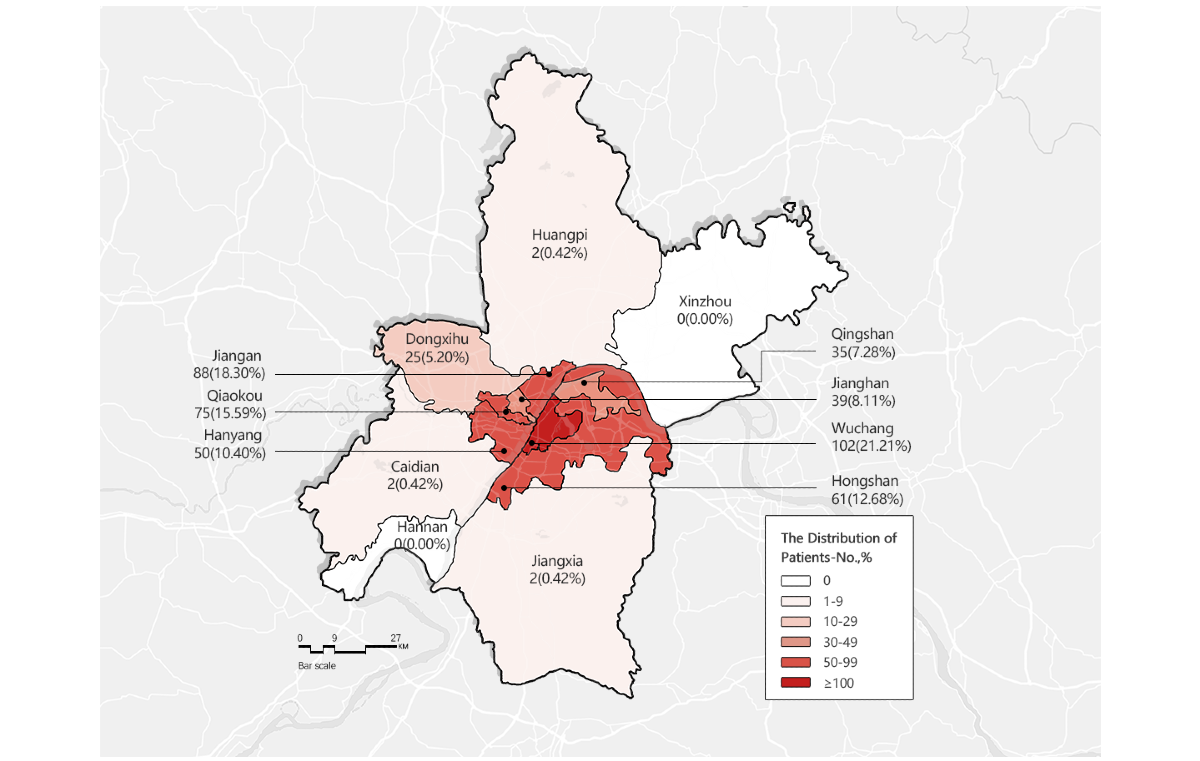
The distribution of patients throughout Wuhan.

**Figure 5:**
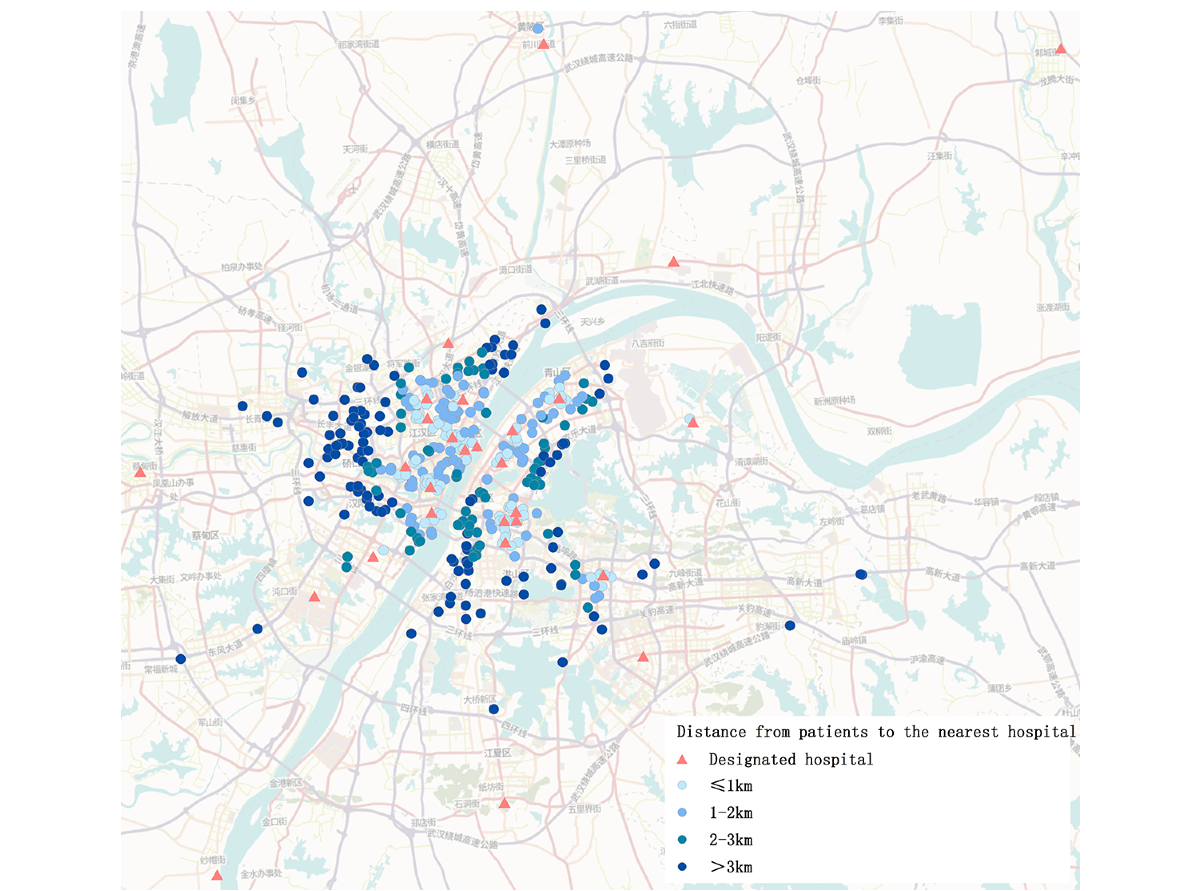
The distance between patients and their nearest designated hospital.

All patients were located in Wuhan, but more patients lived in the central districts (Hongshan, Jiang'an, Wuchang, Hanyang, and Qiaokou) compared to outskirt districts (Figure 4). We further analyzed the distance between patients and the nearest designated hospital. Among these patients, four had missing home address information. We found that 25.57% (123/481) were within 1 kilometer of the nearest designated hospital, 24.74% (119/481) lived within 1-2 kilometers, 17.46% (84/481) lived within 2-3 kilometers, and 32.22% (155/481) lived more than 3 kilometers away (Table 2 and Figure 5).

**Table 2:** The distance between patients and the nearest designated hospital, as well as the relationship between helpers and patients.

Variable		Count, n (%)
**Distance (n=481)**		
	≤1km	123 (25.57)
	1-2 km	119 (24.74)
	2-3 km	84 (17.46)
	≥3km	155 (32.22)
**Relationship between helper and patient (n=329)**		
	Relative	232 (70.52)
	Friend	38 (11.55)
	Patient themselves	34 (10.33)
	Spouse	14 (4.26)
	Sibling	11 (3.34)

### The Relationship Between Helpers and Patients

We explored the relationship between helpers and patients. During data collection, 156 helpers stated that they were family members of patients, but they did not specify their relationship. The remaining 70.52% (232/329) of helpers were the patients' relatives; 11.55% (38/329) were friends, 4.26% (14/329) were their spouses, 3.34% (11/329) were siblings, and 10.33% (34/329) were the patients themselves (Table 2).

## Discussion

### Principal Findings

This study has shown that patients seeking help on Sina Weibo lived in Wuhan and most of them were elderly. Our statistical analysis of the age of patients seeking help on Sina Weibo demonstrated that patients on Sina Weibo were older—the proportion of patients who were ≥65 years was as high as 46.17%. Zhong et al [3] reported that only a small proportion (15.1%) of 1099 laboratory-confirmed COVID-19 patients were aged ≥65 years. On the other hand, our study has found that the highest incidence was among adults over 50 years of age [25].

Additionally, 23.09% of patients had at least one underlying disorder. Fever was the dominant symptom whereas gastrointestinal symptoms were rare. Ground-glass opacity was the most common pattern on chest CT. Among all laboratory-confirmed COVID-19 patients, the most common pattern on chest CT were ground-glass opacity (56.4%) [3]. Our study has shown that the median time from illness onset to RT-PCR testing was 8 days, and the median time from illness onset to online help was 10 days. A recent study showed that the mean time from onset to hospital admission in 44 patients in Wuhan, with onset before January 1, was 12.5 days; in 189 patients with onset from January 1 to 11, the mean time was 9.1 days [5].

Person-to-person transmission of COVID-19 in hospital and family settings has been increasing [26-29]. Family clustering played an important part in increasing the number of COVID-19 cases [30]. Our study provided further evidence of human-to-human transmission, although 60.33% of families had no clustered onset, indicating that home isolation may be effective for patients. However, 39.67% of families had suspected and/or laboratory-confirmed cases among family members. In addition, 36.58% of families had 1 or 2 suspected and/or laboratory-confirmed family members. This is also in line with the finding that patients, on average, transmit the infection to 2.2 other people [5]. Therefore, home isolation might lead to the risk of COVID-19 outbreaks in family clusters [31]. This means that it is crucial to strictly isolate patients and trace and quarantine contacts as early as possible [32,33]. The most stringent centralized medical observation measures should be taken as soon as possible to avoid outbreaks in family clusters due to home isolation [31], such as a modular hospital to treat patients with mild illness [34].

Our research also found that the number of patients in the Wuchang, Jiang'an, Qiaokou, Hongshan, and Hanyang districts was greater than in other districts. Figure 4 shows a central agglomeration of patients; this may be consistent with the outbreak of the epidemic in the Huanan Seafood Wholesale Market in the Jianghan district, which was thought to be the initial infection site from an animal source in China [35] or it may be related to the developed economy, convenient transportation, and the population density in the city center. Therefore, close contact with family members and actual population movements from the outbreak source were risk factors for the spread of SARS-CoV-2.

In total, 32.22% (155/481) of patients lived more than 3 kilometers away from their nearest designated hospital. According to Baidu maps, adults can walk 4 kilometers in 1 hour. Considering that the patients in this study were older and their health condition may have slowed them down even more, we estimate that patients could walk 3 kilometers in a 1-hour period. Hence, this indicates that a patient would need to walk more than 1 hour to see a doctor since public transportation was suspended at the time. This may be one of the reasons why patients wanted to be admitted to a hospital. In addition, on February 5th, the Wuhan municipal health commission designated 28 hospitals for the treatment of laboratory-confirmed patients with the SARS-CoV-2 infection. The empty bed rate of hospitals within the city was only 3.6%. Thus, patients could not be hospitalized for the various reasons above. This also reflected an insufficiency of medical resources during the initial outbreak [36].

We also explored the relationship between helpers and patients. Judging from the content of Sina Weibo posts asking for help, “Mom” and “Dad” were high-frequency words; 70.52% (232/329) of helpers were the patients' relatives, indicating that the publishers of the help information were mostly the children of the elderly. Unfamiliarity with new technology may have hindered elderly people from seeking assistance from the outside world.

With the rapid and effective dissemination of help information, since February 5th, the People's Daily has launched an all-media operation to provide online help channels for patients with the SARS-CoV-2 infection. The government implemented a policy to maximize hospital admissions, which led to a rapid decrease in the number of people seeking help on Sina Weibo on February 6th and remained at low levels since February 8th, indicating that the needs of the public had been met. This also means that it is important to establish new and effective communication mechanisms for the dissemination of important factual information in a timely manner. Through this epidemic, we can see that medical resources are insufficiently allocated. There are substantial regional disparities in health care resource availability and accessibility in China [37]. The rapid increase in the number of patients during the initial outbreak led to a relative shortage of medical resources, which may threaten people with poor self-help capabilities such as the elderly. The government and health departments should pay attention to the elderly population during the outbreak. Social media can be used to understand public demand and aid the government in formulating accurate countermeasures following public demands for help. Although social media can establish effective communication channels, this technology may require a certain threshold, so the government should continue to increase the availability and accessibility of the network to better respond to public health emergencies.

### Limitations

Our study has some limitations. First, given that our data was collected from a social media platform, the description of patients’ symptoms and laboratory information were likely to be incomplete. Second, the urgent timeline for data extraction and the subjective judgment of the collectors might undermine the data quality to a certain extent. Finally, we learned that most of these patients have been admitted to the hospital with government help and many patients remain in the hospital, so we did not compare the 28-day rate for the composite endpoint.

### Conclusions

In summary, our study found that the distance between patients and hospitals and the closure of public transportation further increased the difficulty of hospitalization for the elderly. We recommend the application of centralized medical observations to avoid the spread of COVID-19 through family clusters. In a public health emergency, making full use of available social media platforms can establish effective, factual communication channels and shorten admission times, helping patients get early attention during the Wuhan lockdown. These findings can help the government and health departments pay attention to the elderly population during the outbreak and accelerate emergency responses following public demands for help.

## Abbreviations

API: application programming interface
COVID-19: coronavirus disease
CT: computed tomography
MERS-CoV: Middle East respiratory syndrome
RT-PCR: reverse transcription-polymerase chain reaction
SARS-CoV: severe acute respiratory syndrome coronavirus
SARS-CoV-2: severe acute respiratory syndrome coronavirus 2
